# Multi-tract percutaneous nephrolithotomy combined with EMS lithotripsy for bilateral complex renal stones: our experience

**DOI:** 10.1186/s12894-017-0205-7

**Published:** 2017-02-28

**Authors:** Taisheng Liang, Chenming Zhao, Gang Wu, Botao Tang, Xiangdong Luo, Shangguang Lu, Yu Dong, Huan Yang

**Affiliations:** 1 Department of Urology, Ruikang Hospital Affiliated to Guangxi University of Chinese Medicine, Nanning, China; 2 Department of Urology, Tongji Hospital, Tongji Medical College, Huazhong University of Science and Technology, Wuhan, China

**Keywords:** Bilateral complex renal stones, Multi-tract, PCNL

## Abstract

**Background:**

The treatment of bilateral complex renal stones is a tough challenge for urologists. This study aimed to evaluate the efficiency and safety of bilateral ultrasonography-guided multi-tract percutaneous nephrolithotomy (PCNL) combined with EMS lithotripsy for the treatment of such cases.

**Methods:**

Twenty-seven patients suffering from bilateral complex renal calculi underwent t bilateral multi-tract PCNL. The PCNL began with the establishment of percutaneous nephrostomy access, which was achieved under ultrasound guidance followed by stone fragment and removal by EMS lithotripsy. The same processes were then performed on the ipsilateral and contralateral renal units until the operation terminated. Sheaths left in situ to provide the tracts for the two-stage and the three-stage PCNL procedures. Peri- and postoperative clinical data were collected and analysed.

**Results:**

Renal stones of both sides were completely cleared within three PCNL sessions in 24 cases. Among them, four, thirteen, and seven cases underwent single, second-stage and third-stage procedures, respectively. The total stone-free rate was 88.9%. Three patients failed to receive complete stone clearance. Mean operation time was 78.7 (26–124) min, the mean estimated blood loss was 97.3 (30–250) ml, and the mean length of hospital stay was 18 (10–31) days. No patient required blood transfusion and postoperative fever occurred in 6 cases. Within the follow-up period, stone recurrence occurred in 6 patients.

**Conclusions:**

Ultrasonography-guided multi-tract PCNL using EMS is an efficient method for the treatment of complex renal calculi. According to our experience, it is safe to make multiple tracts on both sides simultaneously.

## Background

Complex renal stones usually refer to staghorn calculi, multiple stones or those associated with anatomical or functional abnormalities. Due to the complicated etiological factors, large stone burdens, high operation risks and high recurrence, it is always a challenge for surgeons to treat such stones, especially those occur in bilateral kidneys. Since Fernström first described percutaneous nephrolithotomy (PCNL) under radiological control in 1976, it has been the standard method for the treatment of large renal stones [1]. Complex stones often require multiple nephrostomy tracts, long operating time, and repeated procedures, which are associated with more access-related complications such as bleeding, infection and deterioration in renal function. Nevertheless, according to the current guidelines, PCNL is still the first-line choice for the management of large or staghorn renal stones [2, 3]. Owning to developments in the technique, it is possible to safely perform multi-tract PCNL on bilateral kidneys at one time. Over the past 4 years, we performed 27 bilateral multi-tract PCNL procedures for the treatment of complex renal stones; therefore, we evaluated the safety and efficacy of this approach.

## Methods

Between October 2011 and November 2015, 27 patients (12 males and 15 females) with bilateral complex stones were admitted to our department. The mean age was 47.8 (41–63) years. All patients had history of renal stones ranging between 6 and 17 years. Eight patients had been treated by operations, five on one side, and three on both sides. All stones were detected by abdominal computerized tomography (CT), and plain film of kidney-ureter-bladder (KUB) as bilateral staghorn or multiple stones. The stone size varied from 2.5 to 8.6 cm. Three cases were associated with moderate to severe unilateral hydronephrosis and nine cases with moderate to severe bilateral hydronephrosis. Urological anatomical abnormalities were observed in five patients: three with ureter pelvic junction obstruction (UPJO) and two with duplex renal pelvis.

**Fig. 1:**
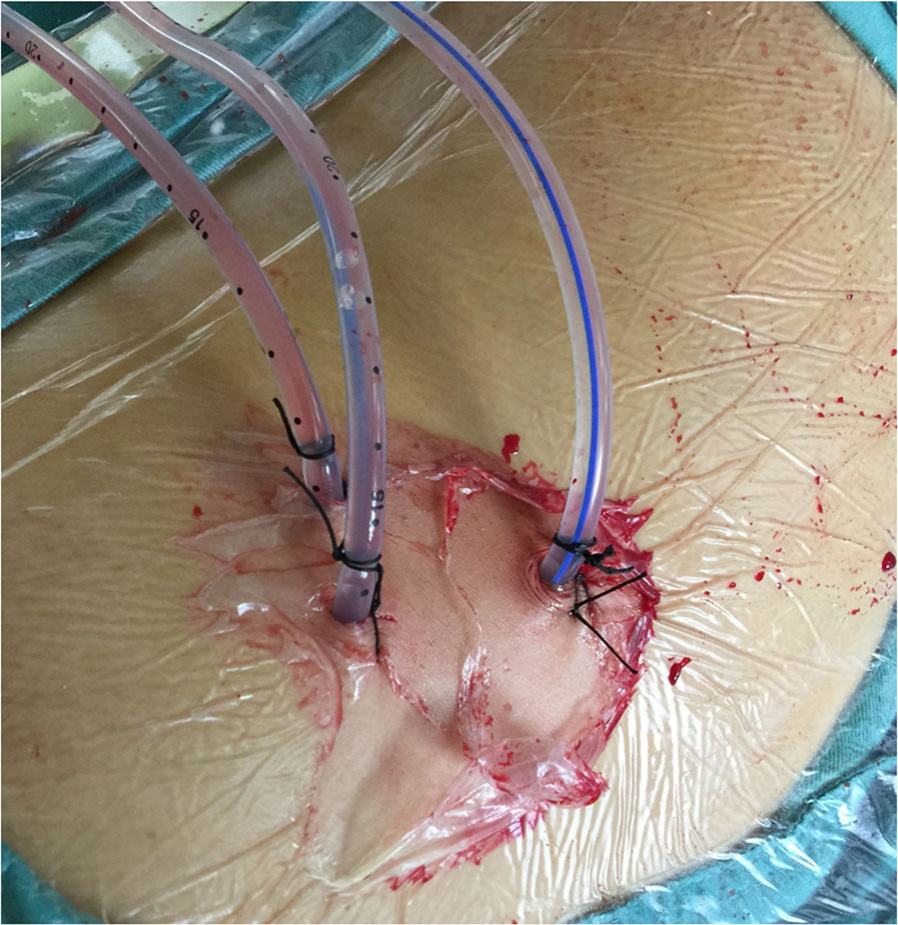
The construction of multiple tracts

PCNL procedures and other aspects of patient treatment mentioned were standard practice. The PCNL procedures were performed under general epidural anaesthesia. A 6-Fr external ureteral catheter was inserted retrograde into the renal pelvis in a lithotomy position for retrograde saline injection if intraoperative artificial hydronephrosis was needed. Then, the patient was placed into the prone position with the renal region elevated. The appropriate percutaneous puncture was achieved under the guidance of ultrasonography (FCUBE9, Korea) using an 18-gauge needle (Urovison, Germany). The first tract was the one that led straight to the maximal stone burden, but stayed away from renal columns, usually through the posterior intermediate calyx. Afterward, the guidewire was inserted into the pelvis or addressed to the target stone. Guided by the wire, tract dilation was accomplished by polytetrafluoroethylene dilators (Urovison, Germany) to 24 Fr. Due to the rigid characteristics of these stones, we applied the pneumatic and ultrasonic endoscopic lithotripter (Electro Medical Systems/EMS-IV) to fragment and remove them. After the stones within the field of vision through the first tract were cleared, the second or third tract was established in the same way to reach those stones in other calyxes. After lithotripsy processes, a 6-Fr double-J stent installed antegrade in the ureter remained for 4 weeks. The operations were terminated by the placement of a 20-Fr nephrostomy tube in each tract. One example of establishment of multiple tracts is shown in Fig. 1. At 5–7 days after PCNL, all patients underwent an X-ray of the KUB to evaluate the residual stones. If necessary, a two-stage or three-stage PCNL operation would be performed through the established tracts. Once the test revealed complete clearance, all nephrostomy tubes were removed. Peri- and postoperative clinical parameters and complications were observed. Clavien-Dindo classification [4] was used to grade the PCNL related complications.

To evaluate the impact from times of PCNL on surgical morbidity, we divided the patients into 3 groups according to the operation sessions. We compared the hospital stay and blood loss among the 3 groups. Statistics analyses were performed using the software IBM SPSS statistics 22. The results were considered significantly different when the *p* value was less than 0.05.

## Results

All 54 kidney units in 27 patients were treated. Percutaneous punctures were performed under the guidance of ultrasonography. The serum creatinine ranged from 123.4 to 667.2 umol/L, and eight patients experienced renal insufficiency. Preoperative urine tests showed that leucocyturia existed in 13 cases, among which *Escherichia coli* was found in six patients. Patients with urinary tract infection were treated with culture-specific antibiotic therapy until the reexamine urine culture was negative. Meanwhile, those with negative urine cultures received empiric antibiotic therapy for 3 days before operation. The patient demographics are shown in Table 1.

**Table 1:** Patient demographics

Number of patients	27
Number of renal unit treated	54
Age, years, mean (range)	47.8 (41–63)
Gender, male/female	12/15
Preoperative body temperature, °C, mean (range)	37.0 (36.5–38.6)
Preoperative serum creatinine, umol/L, mean (range)	204.6 (123.4–667.2)
Mean stone size, cm	2.5–8.6
Urological anatomical abnormalities	Ureteropelvic Junction Obstruction (UPJO) 3
	Double renal pelvis 2
Other relevant conditions	Obesity 3
	Spine deformation 1
Previous stone surgeries	One side 5
	Both sides 3

**Fig. 2:**
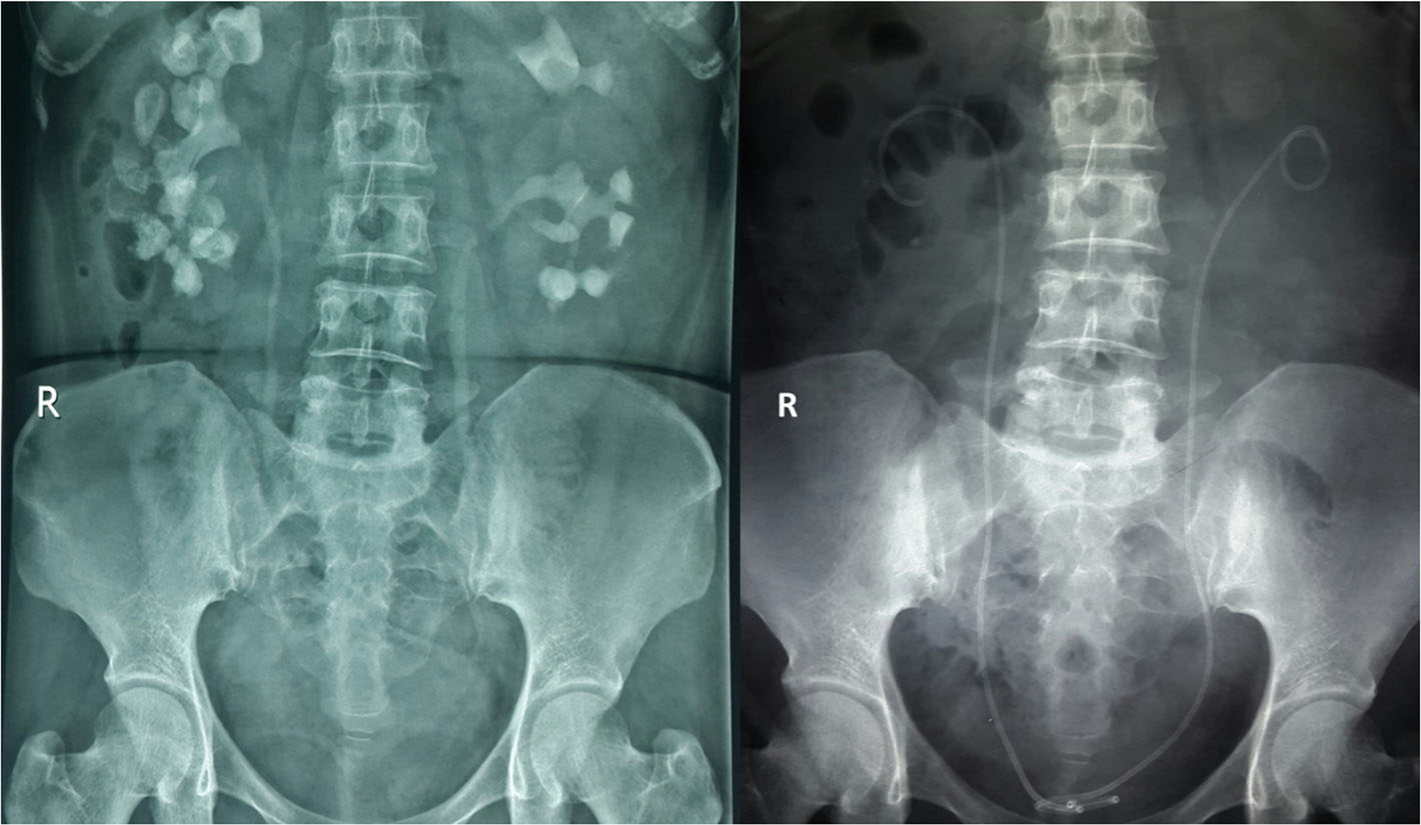
The comparison of the KUBs before and after PCNL of one patient with bilateral complex renal stones

Complete clearance on both sides was achieved in 24 patients. The x-ray film of one example as complete clearance is shown in Fig. 2. Among them, four patients needed one single surgery session, 13 patients needed a secondary PCNL, and three sessions of PCNL were needed in seven patients. Three patients did not achieve complete clearance although one underwent two-stage and two underwent three-stage multi-tract PCNL. The total stone-free rate within three operations was 88.9%. The mean operation time was 78.7 (26–124) min. During surgeries the estimated blood loss was 97.3 (30–250) ml. Severe bleeding occurred in two patients due to mucous membrane injury and tearing of the calyx neck, although no patients required blood transfusion. Six patients had postoperative fever, and two were confirmed to have urinary infection. To prevent possible septic shock, patients with fever were treated with broad-spectrum antibiotics for 3 days, until body temperature and blood tests returned to normal. No patients suffered symptomatic hydrothorax or other visceral organ injury. The Clavien-Dindo classification was assessed as grade II in 6 patients because of the application of antibiotic, whereas the other 21 patients were assessed as grade I. The mean duration of hospital stay was 18 (10–31) days for total cases.

After leaving hospital, all patients accepted follow-up care for 20 (2–42) months. During the follow-up period, two treated kidney units progressed to nephrarctia. Serum creatinine increased by 13.4–132.6 umol/L in three patients compared with the level before surgery; however, for the other patients, serum creatinine decreased to or remained at a normal level. The mean serum creatinine was 116.8 (72.5–154.3) umol/L. Six patients experienced stone recurrence, which required further PCNL or extracorporeal shock wave lithotripsy (ESWL) combined with medical expulsion treatment. All surgical outcomes are shown in Table 2.

**Table 2:** Operative characteristics and results

Tracts in one renal unit, mean (range)	1.8 (1–4)
Number of patients who underwent different sessions of PCNL	One session 4
	Two sessions 14
	There sessions 9
Operation time, min, mean (range)	78.7 (26–124)
Estimated blood loss, ml, mean (range)	97.3 (30–250)
Hospital stay, days, mean (range)	18 (10–31)
1 session	11.8 (10–13)
2 sessions	14 (10–18)
3 sessions	27.7 (24–31)
Complete stone clearance rate within 3 sessions	24 (88.9%)
Postoperative serum creatinine, umol/L, mean (range)	116.8 (72.5–154.3)
Complications	Bleeding 2
	Fever 6
	Infection 2
Clavien-Dindo Classification, Grade (Number)	I (21); II (6)
Follow-up, months, median (range)	20 (2–42)
Recurrence	6
Nephrarctia	2

The statistical analyses revealed that, there was no significant difference in terms of estimated blood loss among the 3 groups (*p* = 0.083). However, more sessions of surgeries were associated with longer hospital stay (*p* < 0.001).

## Discussion

Complex stones are extremely harmful to kidneys because they cause infection, atrophy, renal failure and cancerization. For treatment, multiple tracts are always required to achieve complete clearance and to avoid a second-look procedure such as ESWL or retrograde intrarenal surgery (RIRS). However, multiple tracts are often associated with a higher risk of bleeding [5]. In addition, multi-tract PCNL is very difficult to learn and requires much experience, despite the improvements in devices and techniques over past decades [6]. Some studies have suggested that open or laparoscopic stone surgery is an efficient method for the treatment of complex kidney stones, and is reported to be associated with higher one-session stone-free rate [7, 8]. However, PCNL can be repeated, whereas open or laparoscopic surgery would be increasingly difficult after the first procedure. Moreover, bilateral renal stones are difficult to be treated together in a single session by open or laparoscopic surgery. By contrast, in Cho’s study, multi-tract PCNL had similar safety and effectiveness as conventional single-tract PCNL in patients with complex stones [9]. The AUA guideline also suggested that PCNL with multiple tracts was a safe and effective way for treating staghorn stones, with monotherapy stone-free rate of 79%, and acute complication rate of 15%[3].

A safe and efficient PCNL always begins with successful punctures, especially for complex stones. In our study, no severe complications occurred in any of the 27 patients, although we treated bilateral kidneys for each patient at a single time. We established the tracts under the guidance of ultrasonography. Compared with fluoroscopy, ultrasonography has advantages as it is more convenient without any radiation and it can present the three-dimensional structure of kidneys by turning the probe [10]. Based on our experience, ultrasonography could clearly show hydronephrosis calyces as well as anatomical abnormalities. All tracts could be accurately placed in the line collecting the calyx neck middle and the corresponding calyceal fornix middle. Thus, the tracts provided the least traumatic and most direct access for the fragment of calculi. At the same time, the injury to adjacent viscera was avoided. Moreover, the whole procedure was performed under ultrasonography until dilation was complete, and it was confirmed whether there was any sign of bleeding. It is possible to totally avoid severe bleeding as long as precise punctures are achieved.

Multi-tract PCNL is still controversial. The major area of criticism has been safety during the process, as more than one tract dilatation is considered to be associated with a higher complication rate. Except for a higher risk of bleeding, there might be a significant rise in serum creatinine and drop in creatinine clearance after multi-tract PCNL [11], which means that multiple tracts might cause damage to renal function. However, in our study, only three patients experienced increased postoperative serum creatinine, and based on other studies, there was no evidence to indicate that multiple tracts led to renal insufficiency [12–14]. Moreover, in a number of studies, when compared with single-tract PCNL, no significant difference was reported in terms of complications [9, 11]. In our study, no patients suffered severe perioperative and postoperative complications, and no patients required blood transfusion, showing that more than one tract in a single operation is not always accompanied by high risk. In Singla’s study, the maximal number of tracts used in a single renal unit could be up to six with acceptable morbidity [15]. However, we have to admit, the safety should necessarily be the first principle when we treat complex stones, especially for bilateral stones. Although bilateral PCNL is more efficient and can be performed in both children and adults, it carries a higher rate of overall complications, such as fever, persistent pain, acute renal failure and vomiting, than unilateral PCNL [16]. In addition, once there is hematuria or leucocyturia after bilateral surgery, we cannot determine which is the disease-causing kidney. In consideration of these risks, we performed a careful assessment for every patient before operation to ensure that bilateral PCNL could proceed smoothly. We kept the operation time within 2 h and always started in the renal unit that seemed easier to treat, so that the risk of sepsis was controlled. For each patient, the first tract is the main one, through which we could clear most of the stone burden and relieve the obstruction. Once any severe bleeding or pyonephrosis was found, the operations were stopped immediately, and the tracts remained for the secondary procedure. On the other hand, we acknowledge that more sessions of surgery would inevitably prolong the mean hospital stay, which means more financial cost and higher risk of nosocomial infection, because of the necessary preparation before each session of PCNL, particularly for those with urinary infection. Given these factors, more experience is required to improve the efficiency without any decrease in safety.

For the fragmentation of complex stones, we used the fourth-generation EMS, applying pneumatic and ultrasonic energy together. The stone fragmenting and removal can be carried out at the same time through vacuum suction. The normal saline solution was hung approximately one metre higher than the renal location, which poured naturally as the washing flow, instead of using a pump. Combined with the suction system, a clear surgical field was provided with a low intrapelvis pressure. Using this device, the operation time was shortened and the safety together with efficacy was increased. In our study, only six patients developed postoperative fever, but recovered quickly. In Wang’s study, EMS was even safe for the treatment of calculous pyonephrosis, which was considered as a contraindication of PCNL [17].

Even though most patients also needed more than one stage of operation, multi-tract PCNL significantly improved the clearance efficacy. The totally stone-free rate was 88.9% within three sessions of PCNL, while the recurrence rate was 6/23 within 42 months. Complex stones often result from inherent existing factors such as infection, metabolic disturbance or anatomical abnormality, which occurred in five patients in our series, indicating that prevention is a much more important and difficult task for urologists. Although multi-tract PCNL is an appropriate option to clear complex stones from kidneys with anatomical abnormality, it is of limited value to reduce the recurrence risk. Perhaps, the concomitant of laparoscopic and endoscopic treatment would be the further direction for the treatment of such cases [18, 19].

## Conclusion

Ultrasonography together with pneumatic and ultrasonic endoscopic lithotripter is a widely used device in China for PCNL. With their help, multi-tract PCNL is an efficient and safe method for the treatment of bilateral complex renal stones. Given the limitation of the sample size and the retrospective nature of our study, more multi-center, randomized control studies with large sample size and high quality are required.

## Abbreviations

PCNL: Percutaneous nephrolithotomy
EMS: Electro Medical Systems
CT: Computed Tomography
KUB: Kidney-ureter-bladder
ESWL: Extracorporeal Shock Wave Lithotripsy
RIRS: Retrograde intrarenal surgery
